# Site Specific Cleavage Mediated by MMPs Regulates Function of Agrin

**DOI:** 10.1371/journal.pone.0043669

**Published:** 2012-09-11

**Authors:** Trushar R. Patel, Georgina Butler, Ainsley McFarlane, Irene Xie, Christopher M. Overall, Jörg Stetefeld

**Affiliations:** 1 Department of Chemistry, University of Manitoba, Winnipeg, Manitoba, Canada; 2 Centre for Blood Research, University of British Columbia, Vancouver, British Columbia, Canada; Zhejiang University School of Medicine, China

## Abstract

**Background:**

Agrin is the key inducer of postsynaptic differentiations at the neuromuscular junction. The multidomain heparan sulfate proteoglycan is mediating via its N-terminal segment the interaction with laminin, whereas the C-terminal portion is responsible for Dystroglycan binding and clustering of the Acetylcholine receptor. Matrix metalloproteinases (MMP) are known to play essential roles in matrix remodeling, degradation and regulation of extracellular signaling networks.

**Principal Findings:**

Site-specific processing of Agrin provides key insight into regulatory effects of Matrix metalloproteinases (MMPs). Here, we present a detailed study of agrin processing by different MMPs together with a molecular understanding of binding and cleavage at both terminal fragments. The data suggest for a regulatory effect of MMP cleavage at particularly important functional sites of agrin. Cleave of agrin abolishes the agrin-laminin complex formation and the Acetylcholine receptor clustering at the neuromuscular junction.

**Conclusion/Significance:**

Agrin is a target of specific MMP processing resulting in agrin subfragments with different regulatory activities. MMP processing is a powerful tool to regulate extracellular signaling networks.

## Introduction

Agrin is a heparan sulfate proteoglycan best known for its function to induce and maintain postsynaptic specializations at the neuromuscular junction (NMJ), the clustering of antigen-specific T cell receptors at the I-synapse and the promotion of axon outgrowth in the central nervous system (CNS) [1]. The gene encoding agrin undergoes alternative mRNA splicing at several sites, resulting in protein isoforms that differ in expression, localization and function [2]. Agrin induces the aggregation of acetylcholine receptors (AChRs) via LRP4 mediated binding on the postsynaptic membranes of muscle cells, at sites juxtaposed to the presynaptic nerve terminals [3]. These specializations ensure an efficient and timely response of the muscle to the neurotransmitter acetylcholine that is released from the nerve into the synaptic cleft.

Agrin is a multidomain mosaic protein with key functional sites at the very N –and C-terminus. The agrin - laminin interaction is required for the localization of agrin to the synaptic basal lamina as well as other basement membranes. The high-affinity interaction of agrin with the coiled-coil domain of laminin is mediated by the N-terminal agrin (NtA) domain [4], [5]. Structural studies of the NtA revealed an oligosaccharide/oligonucleotide-binding fold (OB-fold) with several possible sites for interaction with different ligands [6]. A structurally-guided site-directed mutagenesis approach to map the laminin-binding site of NtA has facilitated the development of a model in which the NtA globular β-barrel and the laminin fibrillar coiled coil form a hetero-tetrameric coiled coil that results in a very tight non-covalent complex [7], [8]. Induction of AChR clustering at the muscle cell surface by agrin is mediated via its C-terminal G3 domain—clustering *in vitro* and *in vivo* requires specific inserts of 8, 11 or 19 residues at the B splice site within the G3 domain and the presence of calcium. Another function of agrin is the binding to α-dystroglycan (α-DG), a peripheral membrane protein that is tightly associated with the transmembrane β-dystroglycan derived from a common precursor protein by posttranslational cleavage. Binding of agrin to α-DG requires both the G2 and G3 domains and is Ca^2+^ -dependent.

The importance of agrin is underlined by its multi-functional induction of the postsynaptic apparatus and the mechanical maintenance of the synapses *in vivo*. Agrin-deficient mice lack postsynaptic specializations and die at birth of respiratory failure. Severe congenital muscular dystrophies such as MDC1A are characterized by the failure to form the laminin scaffold, and the missing interaction with α-DG. Ruegg et al have shown that a miniaturized version of agrin (miniagrin) could restore muscle function [9]. Whereas the NtA domain mediates the agrin-laminin-8 interaction, the G2–G3 tandem (A_0_B_0_) stabilizes the binding to α-DG. Miniagrin thus serves as a successful example of functional compensation via miniaturization.

In addition to alternative splicing, the functions of proteins and the domain structure of modular proteins can be altered by proteolytic cleavage. Proteolysis can release domains with novel functions, destroy or alter the activity of the original protein, or alter the localization, interactions and hence functions of the protein. Matrix metalloproteinases (MMPs) are a family of 23 extracellular zinc-dependent endopeptidases that regulate and fine-tune many physiological processes by cleaving a wide range of substrates to increase, decrease, or switch their activities [10]. This processing can be as subtle as trimming 4 residues from the N-terminus of chemokines, to the shedding of whole receptor ectodomains from the cell membrane, or the release of growth factors from their binding proteins [11]. Many MMP substrates are components of the extracellular matrix and their cleavage facilitates extracellular remodeling [12], [13]. Yet the processing of extracellular matrix molecules is not limited to degradation—there is a precedent for MMP generation of molecules with unique functions from modular matrix proteins such as fibronectin [14] and collagen type IV [15]. MMPs play central roles in morphogenesis, wound healing, tissue repair, remodeling in response to injury, and in progression of diseases such as atheroma, arthritis, cancer and chronic tissue ulcers. The four tissue inhibitor of matrix metalloproteinases (TIMPs) are the major endogenous regulators of MMP activities, and are therefore important regulators not only in matrix turnover but also in cellular activities [16], [17]. Remarkably, the OB-fold structure of the NtA is striking similar to the inhibitory N-terminal domain of TIMP-1 [6], suggesting a potential MMP-inhibitory function of the NtA.

Changes in the distribution of agrin during synaptic remodeling, denervation and reinnervation reveal that agrin can be quickly and efficiently removed from axon termini. MMP-3 has previously been shown to cleave and remove agrin from the synaptic basal lamina and to process neuronal agrin following ischemia [18]. Regulated inhibition of MMP-3 results in changes in structure and function of the neuromuscular junction [19], [20]. In addition, agrin was implicated as a substrate of MMP-14 in a degradomic screen [21]. However, it is not clear whether other MMP's can process agrin, nor where the processing might occur. Agrin remains associated for several weeks with the synaptic basal lamina *in vivo* and mice that overexpress NtA-containing agrin at the NMJ show increased stability of postsynaptic structures. To shed light on these controversial findings, we performed for the first time an in-depth characterization of MMP-specific processing of agrin. The data reveal that agrin does not inhibit the activity of MMPs. In contrast, agrin is a target for MMP-specific processing at points of strategic importance to its structure and function.

## Materials and Methods

### Expression and purification of NtA-Fs and miniagrin

The pCEP-Pu plasmids containing the NtA-Fs (NtA domain plus follistatin-like domain) and miniagrin (NtA domain plus follistatin-like domain followed by all three globular C-terminal domains (G1, G2 and G3) interlinked by EGF-like repeats) gene were used for eukaryotic expression of miniagrin as described earlier [22], [23]. We established a stably transfected HEK 293 cell line to obtain NtA-Fs and miniagrin proteins using the non-liposomal lipid transfection reagent Effectene™ (Qiagen, California, USA), using the protocol described by the manufacturer. HEK293 cells were grown in Dulbecco's modified Eagle's medium (DMEM) with 1% glutamine, 10 mM sodium pyruvate, 10% fetal bovine serum (FBS), 100 µg/mL of penicillin and 100 µg/mL of streptomycin. Transfected (puromycin resistant) cells were selected using puromycin at a concentration of 2 µg/mL. The stably transfected cells were grown at 37°C to about 80% confluence and were then transferred to expression medium (growth medium without FBS). Conditioned medium containing secreted proteins was collected at regular intervals and replaced with fresh expression media. The conditioned medium was centrifuged at 2,000×g for 5 min to pellet the cells before storing at −20°C. After thawing, medium was dialyzed overnight against dialysis buffer 1 (50 mM Tris, pH 7.5, 200 mM NaCl) at room temperature and then concentrated using a membrane filter with a molecular weight cut-off of 30 kDa. Both proteins were purified to homogeneity by affinity chromatography using a Ni^2+^ column (GE Healthcare, USA). Several 1 mL fractions were collected from the column and analyzed by Tricine SDS-PAGE (15% for NtA-Fs and 9% for miniagrin). Pooled fractions were then dialysed at room temperature against dialysis buffer 1 containing 10 mM EDTA. EDTA was removed by a third dialysis step at room temperature against buffer 1. The concentration of purified proteins was calculated from the measured absorbance at 280 nm, using a molar extinction coefficient of 86980 M^−1^ cm^−1^ for miniagrin and of 20815 M^−1^ cm^−1^ for NtA-Fs from the ProtParam tool available on the ExPASy server [24]. Purified proteins were then concentrated using a 10 kDa and 50 kDa-cutoff membranes for NtA-Fs and miniagrin respectively and stored at 4°C.

### Investigation of inhibition of MMPs by NtA-Fs/miniagrin

Six different MMPs – MMP-2, -3, -7, -12, -13 or -14 (various concentrations from 0.8–6 nM) were incubated (triplicates) for 2 hours at room temperature with either buffer alone (0.1 M Tris-HCl pH 7.5, 10 mM CaCl_2_, 0.1 M NaCl, 0.05% Brij35) or with 50 nM TIMP-1 or 1 µM of miniagrin. The quenched fluorescent substrate Mca-PLGL-Dpa-AR-NH2 was added to final concentration of 1 µM and cleavage was monitored in a POLARstar OPTIMA (BMG Labtech, USA) at 320 nm excitation and 390 nm emission wavelengths. The rate of substrate cleavage was normalised against the rate obtained for each MMP incubated with buffer alone.

### Processing of NtA-Fs and miniagrin by MMPs

In order to investigate whether any MMPs process NtA-Fs and miniagrin, both proteins were incubated for 18 hours at 37°C at a 10∶1 molar ratio with MMP-1, 2, 7, 8, 9, 12 (catalytic domain), 13 or 14 (soluble form). Since MMP-3 has been shown previously to remove agrin from basal lamina, a range of concentrations of MMP-3 (1∶250, 1∶100, 1∶50 and 1∶10 molar ratio) were incubated with miniagrin. For control experiments, NtA-Fs/miniagrin was incubated with buffer only (0.1 M Tris-HCl pH 7.5, 10 mM CaCl_2_, 0.1 M NaCl) at 37°C for 18 hours. After incubation, all samples were analysed by either 13% or 9% Tris-Glycine or 15% Tris-Tricine SDS-PAGE that were stained using silver. Proteins were transferred to PVDF membrane following SDS-PAGE and stained with Coomassie Blue R250. Selected products from PVDF membrane were excised and subjected to Edman sequencing to determine cleavage sites on NtA-Fs/miniagrin (at the Tufts University protein sequencing facility).

### Effect of MMP-12 processing on NtA-Fs/miniagrin-laminin complex formation

The NtA-Fs/miniagrin protein was incubated with MMP-12 at a 10∶1 molar ratio or each was incubated separately for 18 h at 37°C. Mouse laminin-1 (EHS Sarcoma, Sigma-Aldrich, USA) was plated into a 96-well flexible assay plate (BD Falcon™, USA) at 5 µg/mL in Vollers buffer (15 mM Na_2_CO_3_, 35 mM NaHCO_3_ pH 9.6) and incubated for 18 h at 4°C. After 3 washes with phosphate buffered saline (PBS), the plate was blocked with 2.5% BSA in PBS for 90 min. Following further washes, the miniagrin digested with MMP-12 or MMP-12 were added at various concentrations (0.2 to 9.2 µg/mL) in PBS and incubated at room temperature for 3 hours. After 3 washes with 0.05% Tween 20 in PBS, bound miniagrin was detected using mouse anti-his antibody (Qiagen, USA) followed by goat anti-mouse alkaline phosphatase-conjugated secondary antibody (washing with 0.05% Tween20 in PBS). Alkaline-phosphatase activity was quantified using p-nitrophenyl phosphate (Sigma-Aldrich, USA), reading at 405 nm in a Thermomax microplate reader (Molecular Devices, USA).

### Computational Modelling for MMP- 1 and MMP-12 processing of miniagrin

We have designed computational models for the MMP-miniagrin interaction based on homology models. The high-resolution structures homologous for NtA and G2 domains were found using SWISS-MODEL that provided model 1JB3 for NtA [6] and model 1PZ7 for G2 [25] domain from the PDB server. We also selected model 2J0T [26] for MMP-1 and model 2OXU [27] for MMP-12 from the PDB server. Docking of MMP-1 with NtA and G2, respectively, was performed using FTDOCK [28]. The lowest energy docked structures were subject to 500 cycles of unrestrained Powell minimization using CNS [29]. Harmonic restraints were imposed on both the MMP and the NtA/G2 atoms (2 kcal/mol Å^2^) with increased weight (20 kcal/mol Å^2^). Final docking results were visualized using program DINO [30].

## Results

### NtA-Fs and miniagrin do not inhibit the activity of matrix metalloproteinases

For our studies we expressed miniagrin and the isolated NtA-Fs tandem. Both protein versions were purified using affinity chromatography and were analyzed using SDS-PAGE and Dynamic Light Scattering (DLS) experiments (see also Materials and Methods and Figure S1). From these results, it is clear that no higher molecular weight species or degraded protein components are present and that the proteins are highly pure and homogenous and therefore suitable for further experiments.

**Figure 1:**
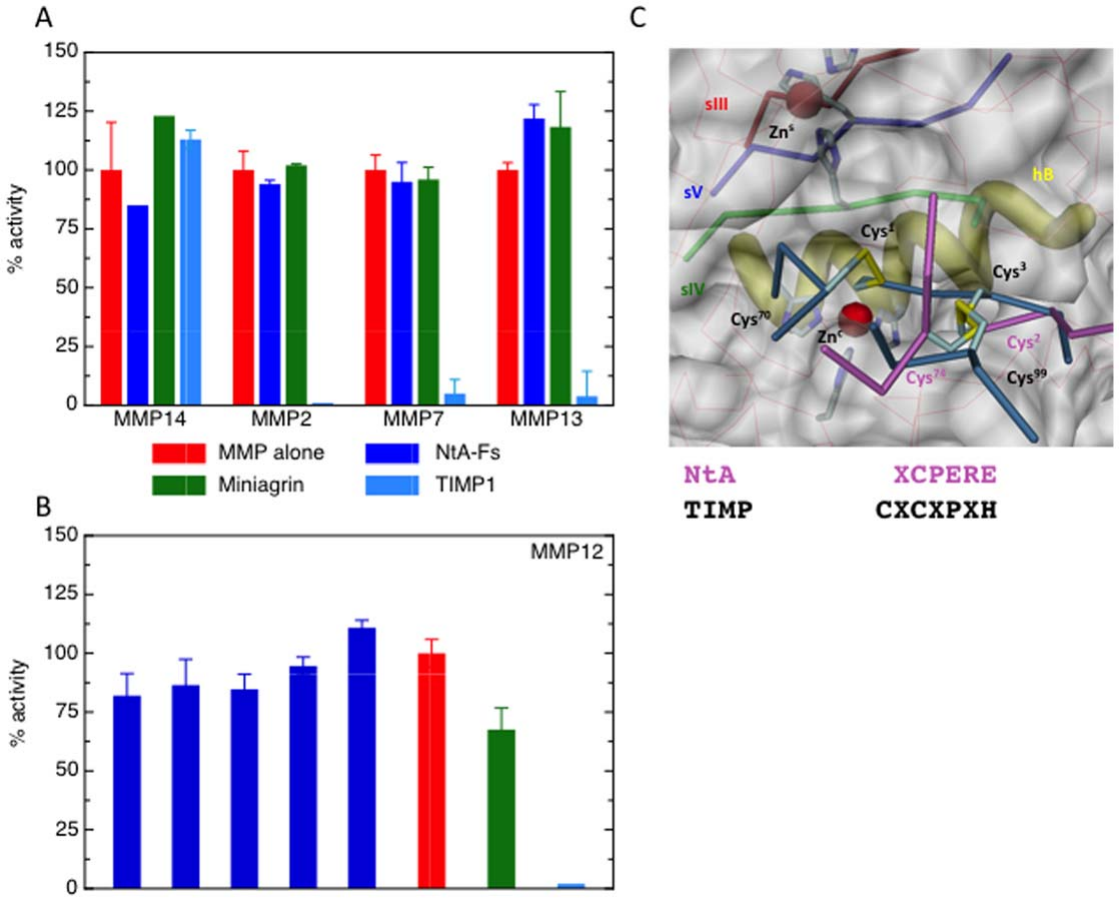
Agrin does not inhibit MMPs. Comparison of inhibitory action of miniagrin, NtA-Fs and TIMP-1 for: (A) MMP-2 (1 nM), MMP-7 (5.6 nM) and MMP-14 (4 nM) and MMP-13 (10 nM) with 50 nM TIMP-1, 100 nM Nta-FS and 1 µM miniagrin; (B) MMP-12 (1 nM) with a range of NtA-FS concentrations, 1 µM miniagrin and 50 nM TIMP-1; Values are expressed as a percentage of the uninhibited MMP activity +/− standard deviation. (C) Superimposition of NtA (Cα-backbone in pink) and TIMP-1 (Cα-backbone in steelblue) projected into the active site cleft of MMP-3 (pdb-code 1uea). Essential elements of MMP are highlighted in different color schemes (sIII-v, hB and both histidine fingers). The key disulfide bridges of TIMP-1 (Cys^1^–Cys^70^ and Cys^3^–Cys^99^) and NtA (Cys^2^–^74^) are labeled accordingly. Structural (Zn^s^) and catalytic zinc (Zn^c^) ions are shown as red spheres. The N-terminal sequences for NtA (in pink) and TIMP-1 (in black) reveal the missing Cys in position 1.

Due to structural similarity between the N-terminal tail of NtA and the inhibitory segment in the N-terminal domain of the endogenous MMP inhibitor TIMP-1, we investigated the potential inhibitory activity of miniagrin and NtA-Fs (NtA domain plus follistatin-like domain) for a selection of MMPs. MMP-2, -3, -7, -12, -13 and -14 were incubated with miniagrin, NtA-Fs and TIMP-1 as reference (Figure 1A–C; data for MMP-3 not shown). None of the MMPs were inhibited by NtA-Fs or miniagrin, whereas, as expected, all except MMP-14 were inhibited by TIMP-1. In addition, neither agrin version had significant effects on proMMP-2 autoactivation *in vitro* nor activation of proMMP-2 by MMP-14 when added exogenously to a HT1080 cell system (data not shown). This lack of inhibitory activity is likely to be due to differences in the N-terminal six residues of TIMP-1 and NtA. The N-terminal ridge of TIMP-1 is constituted by two cysteine residues (Cys^1^ and Cys^3^) that are crucial for interaction with the MMP active site and hence for inhibition. A structural superimposition of both, TIMP-1 and NtA, fitted into the active site cleft of MMP-3 revealed a mechanistic explanation for the non-inhibitory function of agrin (Figure 1D). The missing N-terminal cysteine residue at position 1 of NtA prevents chelation of the active site zinc ion and therefore MMP activity is not inhibited since Asn^1^ is not able to mediate binding of the catalytic zinc. Whereas TIMP-1 has two subsequent disulfide linkages (Cys^1^–Cys^70^ and Cys^3^–Cys^99^) at the N-terminal segment that cross-link two adjacent loop segments, NtA has only one disulfide bridge formed between Cys^2^ and Cys^74^ from loop 3–4. In case of TIMP's, the central disulfide-linked segments bind to either side of the catalytic zinc and Cys^1^ bidentally coordinates this zinc.

### NtA-Fs and miniagrin are processed by various matrix metalloproteases

**Figure 2:**
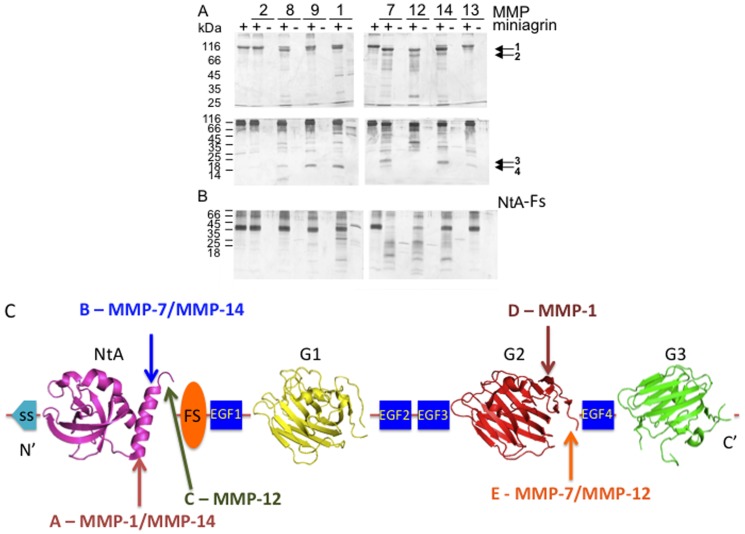
Processing of miniagrin and NtA-Fs by matrix metalloproteases. SDS-PAGE analysis of (A) Miniagrin and (B) NtA-Fs after incubation for 18 h at 37°C alone, or with a 10∶1 molar ratio with MMP-1, 2, 7, 8, 9, 12, 13 or 14 (+). Each MMP was also incubated alone (−). Products were analysed by (A) 9% Tris-glycine (upper panel) and 15% Tris-tricine (lower panel) and (B) 15% Tris-tricine SDS-PAGE with silver staining. Following transfer to PVDF, excised products were subjected to Edman sequencing to determine cleavage sites. (C) Topology scheme of miniagrin with MMP target sites highlighted. Miniagrin is a mosaic protein composed of the following domains: NtA, N-terminal Agrin; Fs, follistatin-like; EGF, EGF–like domains 1–4 and G, globular domains 1–3. Sites A to E correspond to sites detailed in Table 1.

We have selected MMPs from several different families such as collagenases (MMP-1, -8 and -13), gelatinases (MMP-2 and -9), membrane-type MMPs (MMP-14), matrilysin (MMP-7) and metalloelastase (MMP-12) to investigate if they can cleave miniagrin and NtA-Fs (Figure 2). Incubation with MMPs at 37°C for 18 hours followed by SDS-PAGE analysis revealed that miniagrin is processed to some extent by all of the MMPs utilized in the current study, except for MMP-2 (Figure 2A). Prominent cleavage fragments were selected for N-terminal sequencing. The Edman sequencing results obtained for miniagrin cleaved by MMP-1, -7 -12 and -14 are shown in Figure 2C and the cleavage sites derived from these are presented in Table 1. The band immediately below the miniagrin band (indicated by arrow 1 in Figure 2A) reveals cleavages between residues 141 and 162 at the C-terminal α-helix H3 of the NtA domain. This helical element, which contains the major laminin-binding epitope, is the locus for an alternative splice insert encompassing 7 amino acid residues [8]. A slightly smaller band (indicated by arrow 2) has the same N-terminal sequence suggesting additional cleavage(s) at the C-terminus of miniagrin. This was confirmed by sequencing the bands that migrate around 30 kDa on 15% Tris-Tricine SDS-PAGE (arrows 3 and 4 in Figure 2A). N-terminal sequences obtained for C-terminal cleavage products of MMP-1, -7, and -12 indicate processing between the G2 and EGF-4-G3 tandem. Thus, several MMP's can cleave miniagrin at key positions essential for agrin-mediated function in the neuromuscular junction and may regulate agrin function by releasing the NtA and/or EGF-4-G3 domains. Interestingly, the recombinant NtA-Fs tandem was further processed by MMP-7 and -12 and to a lesser extent by MMP-1 and -14, but not by MMP-2, -8, -9 or -13 (Figure 2B). Thus this could suggest differential regulatory mechanisms of MMP activity.

**Table 1:** MMP cleavage sites in miniagrin derived from Edman sequencing data.

MMP/Target –site	Cleavage site (P3-P2-P1* P1′-P2′-P3)	Localization
*N-terminal target sites*		
MMP-1/Asn^141^	(A) L- R-N* L-E-E	Laminin-binding site at the N-terminal portion of NtA Helix H3
MMP-7/Lys^154^	(B) H-R-K* L-L-A	Alternative mRNA splice insert at NtA Helix H3
MMP-14/Asn^141^and Lys^154^	(A)+(B)	see above
MMP-12/Ser^162^	(C) P-N-S* Y-F-T	Linker segment between NtA and Fs domain
*C-terminal target sites*		
MMP-1/His^1822^	(D) E-Q-H* I-R-S	Loop segment after strand s13
MMP-7/Thr^1831^ and MMP 12/Thr^1831^	(E) I-S-T* F-R-A	Linker segment between G2 and EGF4-G3
MMP-3/Thr^1650^ [19]	P-H-T* M-L-N	Concave site of the β-sheet of G2 between β-strands s10 and s11 [25]
Neurotrypsin [39] * β site	V-E-K* S-V-G	Linker segment between EGF4 and G3

* In addition, the authors described also an α-cleavage site, at the SEA domain of agrin, generating a 90 kDa cleavage product [39].

### MMP cleavage of agrin abolishes laminin binding

**Figure 3:**
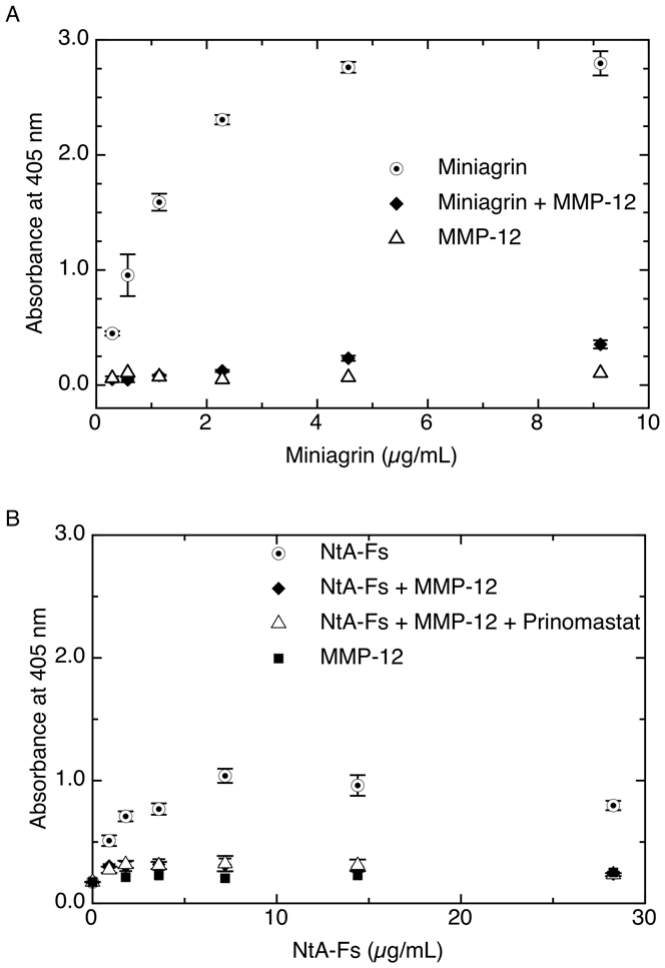
MMP-12 cleavage abolishes agrins capability to bind to laminin. (A) Dose-dependency of binding of miniagrin to laminin-coated plates indicates a specific interaction between full-length miniagrin with immobilized laminin. This interaction is abolished by MMP-12 processing of miniagrin. (B) The dose-dependent binding of the isolated NtA-FS domain to laminin is abolished by MMP-12 cleavage. Incubation with Prinomastat confirms that the MMP-12 does not process the immobilized laminin to reduce NtA interaction. Detection of the polyhistidine tag on the miniagrin and NtA-Fs was carried out with a mouse anti-his antibody, anti-mouse secondary antibody conjugated to alkaline phosphatase and alkaline phosphatase substrate. Colour development was detected at 405 nm.

In order to investigate the implications of processing of miniagrin by MMPs on the interaction of agrin with laminin, we selected MMP-12 that cleaves miniagrin at both the N-terminus and C-terminus potentially generating free NtA and EGF4-G3 domains, respectively. Miniagrin was processed by MMP-12 as shown above and binding to laminin was compared with unprocessed miniagrin using an ELISA-type method (Figure 3A). Briefly, the absorbance at 405 nm for miniagrin increases with increase in miniagrin concentration indicating the interaction of miniagrin with immobilized laminin. However in comparison, when MMP-12-processed miniagrin was studied, the absorbance was significantly reduced suggesting a loss of interaction of miniagrin with laminin due to MMP-12 cleavage. The absorbance for MMP-12 was close to zero at all concentrations as expected. MMP-12 was able to further process recombinantly produced NtA-Fs tandem. Thus we analysed the binding of NtA-FS to laminin following processing by MMP-12 (Figure 3B). As expected NtA-Fs preincubated in the absence of MMP-12 bound dose-dependently to laminin as indicated by an increase in absorbance with increasing NtA-Fs concentration. There was no such increase in absorbance following processing of the NtA-Fs with MMP-12, indicating that laminin binding was abolished by further cleavages of NtA-Fs by MMP-12 as suggested in Figure 2B. The addition of the MMP inhibitor Prinomastat after the pre-incubation of MMP-12 with NtA-Fs, but prior to addition to the laminin-coated plate, confirms that the reduction is due to MMP-12 processing of the agrin rather than of the laminin during the ELISA. In summary, we showed here that the processing of miniagrin by MMP-12 abolishes the interaction of miniagrin with laminin and that MMP-12 eliminates the binding of the NtA domain itself to laminin, probably by further processing of the NtA domain.

## Discussion

In the present study, we have shown for the first time that agrin is specifically cleaved by MMP-1, -7, -12, and -14 at sites of strategic importance for this key organizer of postsynaptic specializations at the neuromuscular junction (Figure 2 and Table 1). The NtA domain is the most highly conserved domain in agrin, and serves to keep agrin at its site of secretion by binding to laminin. This interaction enables agrin to be localized at the synaptic and other basal lamina to support matrix stabilization [5]. In this study, three different cleavage sites were detected within the C-terminal portion of NtA suggesting that the cleavage product remains a stably folded β-barrel. Whereas MMP-1 and MMP-7 have unique targets (sites A and B) inside the C-terminal helix H3 of NtA, MMP-14 overlaps with both recognition patterns (Figure 2C and Table 1). Cleavage sites A and B are of great importance to the function of agrin. Site-directed mutagenesis experiments showed that Leu^142^ (P1′ residue at site A) is one of the three key residues mediating the unique agrin-laminin complex formation [8]. There the β-branched side chain of the leucine fork is involved in van der Waals contacts with solvent exposed alanine residues of the triple helical laminin coiled coil. It is interesting to note that the sequence EHRKLLA that contains the cleavage site B (Lys^154^ at P1 position) for MMP-7 and MMP-14 is the 7 amino acid residue alternative mRNA splice insert only found in agrin versions expressed at motorneurons [31], [32]. Isoforms of agrin without the insert would be immune to removal of the NtA by MMP-7 and MMP-14 and may be subject to different regulation than the splice-insert containing isoforms.

In contrast, MMP-12 cleaved in the linker segment between the NtA and the subsequent Fs domain (site C), but, unlike the other MMPs tested, also seemed to further process or even degrades the NtA-Fs domain to abolish laminin binding. This suggests differential regulation of NtA function by the MMPs, which are in addition secreted by different cell types and under specific conditions, i.e. subject to spatial and temporal regulation *in vivo*. Particularly interesting is the reduced laminin-binding capability of miniagrin after MMP-12 processing. Denzer and coworkers have shown that the NtA domain alters the size of induced AChR clusters but not the overall extent of the induction [33]. AChR aggregates induced by full-length agrin are more than twofold smaller than those induced by a fragment without the NtA domain (equivalent to cleavage products A–C). One explanation for this phenomenon might be that due to its tight interaction with laminin the NtA causes immobilization of small AChR clusters at the basal lamina, thereby preventing their fusion to form larger aggregates or alternatively, prolonging the time taken for fusion. In addition, it has been speculated, that agrin-laminin complex formation is the reason why, after degeneration of the nerve and the muscle, AChR aggregation and agrin-like immunoreactivity remains at former synaptic sites for weeks [34]. Thus, removal of the NtA domain from agrin and differential further processing by different MMPs might be a control mechanism to regulate the networking mediated by laminin-agrin interactions.

Another consequence of MMP-12 cleavage of agrin may be the regulation of binding of different growth factors. Versions of agrin starting with the first Fs domain (equivalent to cleavage site C), can bind to BMP2, BMP4 and TGFβ1 [35]. In contrast, full-length agrin does not show any binding capacity for these three growth factors. Moreover, whereas BMP2 and BMP4 were inhibited, TGFβ1 activity was slightly increased. The growth-factor binding activity of different agrin forms is important for its role in development and maintenance of the NMJ. Regulation of growth factor binding activity of proteins such as IGFBPs, pleiotrophin and connective tissue growth factor by MMP processing is a well established concept, though it has typically evoked a decrease in binding [36], [37]. Therefore, MMP processing may regulate growth factor binding by agrin, and thus growth factor bioavailability – processed versions of agrin may serve as a reservoir of these growth factors, by localizing their action and regulating their growth promoting activity.

At the C-terminus of agrin, all three MMPs (MMP-1, -7 and -12) have unique recognition sites (Table 1 and Figure 2C): Whereas MMP-1 cleaves at the C-terminal end of the G2 domain (site D), MMP-7 and -12 both cleave at site E. Removal of the EGF4-G3 domains is predicted to prevent binding of agrin to α-DG since the interaction requires two G domains, preferably G2-EGF-4-G3. On the other hand, the release of the C-terminal tandem EGF4-G3 by MMP processing may favour the binding of heparin to the G1-EGF2-EGF3-G2 moiety. It is known that LRP4/MuSK binds the G3 domain of agrin and subsequently AChR aggregation occurs. This raises the question as to whether MMP release of the EGF4-G3 domains affects AChR aggregation activity. Scotton *et al*. found that MuSK phosphorylation activity is decreased tremendously if only the G3 domain is present as compared to miniagrin [23]. This suggests that agrin processing at the C-terminus by MMP's might be a control mechanism to limit MuSK phosphorylation, and hence AChR aggregation activity. MMP-3 cleavage at the C-terminal G2 domain (see also Table 1) leads to an accumuluation of agrin at the neuromuscular junction [19]. In addition, MMP-3 null mutant mice show dramatic changes in the ultrastructure of the neuromuscular junction suggesting that MMPs are involved in regulating the size and structure of the postsynaptic apparatus. In agreement to previous reports of MMP-3 activation at neuromuscular junctions and MMP-3-mediated removal of agrin from the synaptic basal lamina, the present study further supports a mechanism for the control of AChR aggregation activity by MMPs. It has been reported that ischemia-induced MMP-3 processes agrin expressed in neurons and that processed agrin is released from tissue membrane fractions to tissue protein extracts [21]. These observations indicate that full-length agrin and agrin domains released by MMP processing may have different biological roles to play. A pathogenic role of MMP-3 dependent cleavage of agrin in neurological disorders has been suggested in addition to being associated with autoimmune diseases [38].

In an independent line of evidence, the synaptic serine protease neurotrypsin has been shown to cleave agrin liberating a 90 kDa (α-site: at the SEA domain) and a 22 kDa (β-site: at the G3 domain) subfragment (see also Table 1) [39]. The cleavage products of the synaptic agrin reveal inhibitory activity on the Na^+^-K^+^-ATPase resulting in membrane depolarization and increased action potential [40]. This finding suggests that the release of active cleavage fragments might promote interactions with different receptor systems abolishing the function of full-length agrin. Furthermore, in glioblastoma, agrin is degraded by MMPs which causes a dramatic effect on the distribution of aquaporin-4 (AQP4) [41]. The agrin mediated architecture of orthogonal arrays of particles (OAPs) is disrupted and the polarity of astroglial cells is affected.

**Figure 4:**
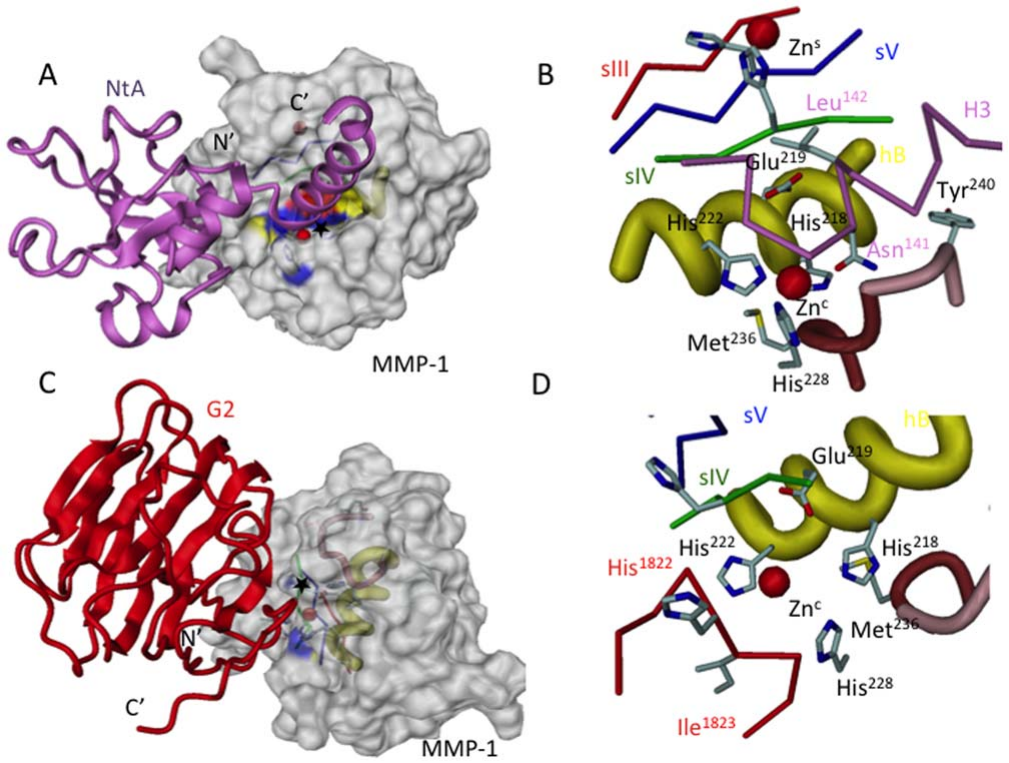
Structural models of MMP-1 cleavage. Overall and detailed view of the MMP-1 in complex with NtA (A, B) and G2 (C,D). The NtA domains are drawn in pink and the G2 domain in red. In (A) and (C) the MMP-1 model is shown in a surface presentation with helix hB highlighted in yellow, the zinc-ligating histidines in blue and Glu^219^ in red. Zn-ions are drawn as red spheres. The cleavage site is marked with *. In (B) and (D) the 1,4 tight “Met-turn” (brown) and “S1′ wall forming segment” (pink) with Met^236^ and Tyr^240^ are highlighted. Amino acid residues in P1 and P1′ position are shown in atom color code.

Modelling studies of MMP-1 with the NtA domain (Figure 4 A and B) and the G2 domain (Figure 4 C and D) as targets reveal insights into their substrate recognition pattern. In case of cleavage site A (Asn^141^), the P1*P1′ substrate residues of NtA bind to the MMP-1 active site cleft in a manner reminiscent of the inhibitory region of TIMP-1 (see also Figure 1D). The linker segment between β-barrel and N-terminal portion of the long C-terminal α-helix H3 fit into the active site cleft of MMP-1 as shown for the N-terminal ridge and helix 1 in TIMP-1 [42]. In its center and to the right site, this helical cleft exhibits a slightly negative potential, which is compensated by the antiparallel orientation of both helical segments, H3 (NtA) towards hB (MMP-1). This orientation defines NtA as a right-side binder, inserting between the antiparallel bulge-edge segment and the parallel S1′ wall-forming segment of the cognate MMP. Leu^142^ at the P1′ position is oriented towards the three-stranded mixed β-sheet composed of strands sIII-sV. Interestingly, this aliphatic residue is part of the laminin recognition motif of NtA and together with Val^149^ forms a unique heteromeric coiled coil assembly with the trimeric long arm of laminin [7], [8]. The fact that helix H3 is not involved in additional interactions with the core of the NtA domain suggests that the cleavage product is a stable OB-fold protein, truncated by the C-terminal α-helix. This finding is supported by CD-spectroscopic data of ΔH3 version of NtA. In contrast to the MMP-1-NtA complex which covers a contact area of 1140 Å^2^, the domain-domain contact in the MMP-1-G2 complex is more pointed and spans a surface area of 680 Å^2^. The final β-strand s13 of the convex β-sheet of the G2 sandwich structure meanders via a short linker segment to the C-terminal disulfide bridge (Figure 4C). This motif extends the jellyroll motif of the G domains and has a large accessible surface area. The P3-P2-P1*P1′ side chains of the substrate are oriented in an antiparallel manner to the active site helix hB allowing a good fit towards the S1′ pocket and the Met-turn segment (Figure 4D). As shown for the MMP-NtA cleavage, also here the cleavage products are most probably stably folded EGF-4-G3 tandems.

In conclusion, all MMPs investigated in this study process agrin within the N-terminal region to release the NtA domain and/or at the C-terminus to release the EGF4-G3 domains. Remarkably, none of the observed cleavage recognition motifs is inside the β-barrel of NtA or the β-sandwich of the G domains. It is likely that by removal of these specific modules, these MMPs regulate the interactions and functions of agrin. Cleavage of agrin by MMPs separates the N-terminal laminin binding portion from the C-terminal moiety that modulates AChR clustering and α-DG binding and may shift the balance between distinct regulatory functions of agrin. As revealed by sonomicrometry studies of *ex vivo* diaphragms after phrenic nerve injury, MMPs are transiently upregulated and have a specific impact on degradation of laminin and agrin, affecting the ECM environment and thereby impairing diaphragm functions [43]. Our findings suggest a novel therapeutic potential for specific inhibition of agrin-specific degradation sites.

## Supporting Information

Figure S1 **Dynamic light scattering profile for miniagrin and NtA-Fs: Single peaks indicate the presence of aggregate-free protein.** The measurements were performed at 1.8 mg/ml for NtA-Fs and at 0.2 mg/mL for miniagrin. Samples were filtered using a 0.1 µm centrifugal filter (Millipore, USA) in a buffer containing 50 mM Tris pH 7.5, 200 mM NaCl. All protein samples were allowed to equilibrate for 4 minutes at 20°C before data collection by DLS. At least four measurements were made and the average value was used in the subsequent calculations. The resulting data were analyzed using DTS software (Version 5.10.2, Malvern Instruments Ltd., Malvern, UK). The hydrodynamic radius (Rh) was measured at different concentrations before extrapolating to infinite dilution.
(TIFF)
