# Supplementary figures and images for: Site Specific Cleavage Mediated by MMPs Regulates Function of Agrin
Source: PLoS One. 2012 Sep 11;7(9):e43669. doi: 10.1371/journal.pone.0043669 (PMC3439447; doi:10.1371/journal.pone.0043669)

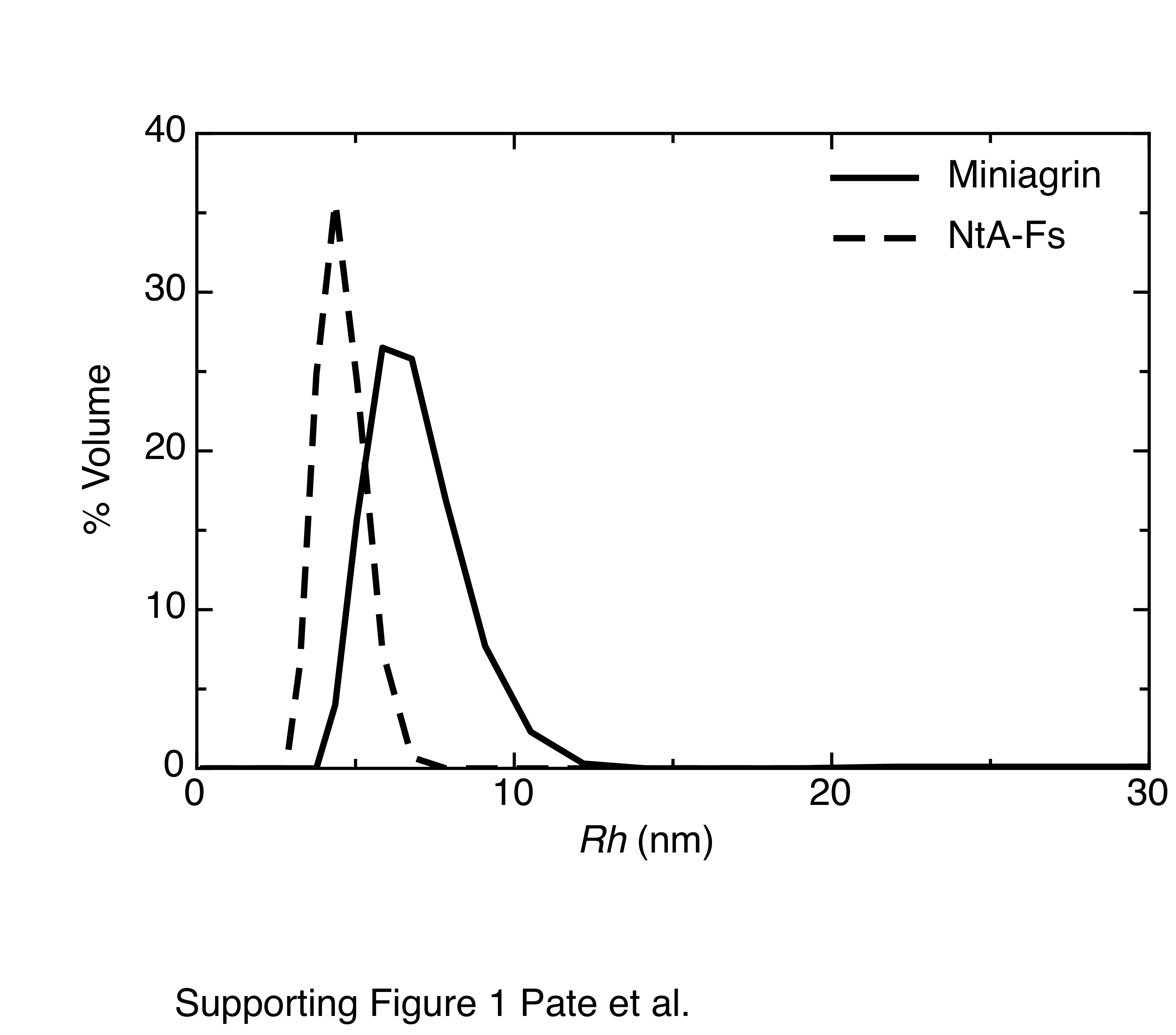

Supplement: Figure S1 — Dynamic light scattering profile for miniagrin and NtA-Fs: Single peaks indicate the presence of aggregate-free protein. The measurements were performed at 1.8 mg/ml for NtA-Fs and at 0.2 mg/mL for miniagrin. Samples were filtered using a 0.1 µm centrifugal filter (Millipore, USA) in a buffer containing 50 mM Tris pH 7.5, 200 mM NaCl. All protein samples were allowed to equilibrate for 4 minutes at 20°C before data collection by DLS. At least four measurements were made and the average value was used in the subsequent calculations. The resulting data were analyzed using DTS software (Version 5.10.2, Malvern Instruments Ltd., Malvern, UK). The hydrodynamic radius (Rh) was measured at different concentrations before extrapolating to infinite dilution. (TIFF) [file pone.0043669.s001.tif]
